# Risk Estimation in Non-Enhancing Glioma: Introducing a Clinical Score

**DOI:** 10.3390/cancers15092503

**Published:** 2023-04-27

**Authors:** Philip Dao Trong, Samuel Kilian, Jessica Jesser, David Reuss, Fuat Kaan Aras, Andreas Von Deimling, Christel Herold-Mende, Andreas Unterberg, Christine Jungk

**Affiliations:** 1 Department of Neurosurgery, Heidelberg University Hospital, 69120 Heidelberg, Germany; philip.daotrong@med.uni-heidelberg.de (P.D.T.);; 2 Institute of Medical Biometry, Heidelberg University, 69120 Heidelberg, Germany; 3 Department of Neuroradiology, Heidelberg University Hospital, 69120 Heidelberg, Germany; 4 Division of Neuropathology, Institute of Pathology, Heidelberg University Hospital, 69120 Heidelberg, Germany; 5 German Cancer Consortium (DKTK), CCU Neuropathology, German Cancer Research Center (DKFZ), 69120 Heidelberg, Germany

**Keywords:** non-enhancing glioma, lower-grade glioma, malignant glioma, IDH mutation, CDKN2A/B, molecular classification, prognostic score, risk estimation

## Abstract

**Simple Summary:**

The preoperative risk estimation of non-enhancing suspected “low-grade” glioma (NEG) is key in determining the optimal timing of diagnosis and treatment to delay malignant progression and avoid undertreatment. The updated 2021 WHO classification brought new facets to glioma grading. Therefore, we sought to identify preoperative risk factors of malignancy in NEG by considering molecular criteria, including IDH mutation and CDKN2A/B deletion status. A total of 72 NEG patients were analyzed, and a high prevalence of malignant gliomas was detected considering both the traditional WHO grading (WHO grade 3 + 4) and the integrated molecular classification (IDH^wt^ glioblastoma WHO grade 4 and IDH^mut^ astrocytoma WHO grade 4). Easily determinable preoperative factors (age, T2/FLAIR mismatch sign, and SVZ involvement) were identified by uni- and multivariate analyses and incorporated into a score. The score estimates the probability of an NEG harboring a malignant glioma. Finally, the score was validated in a cohort of 40 NEG patients and proved to be a better prediction model than the Pignatti score or the T2/FLAIR mismatch sign.

**Abstract:**

The preoperative grading of non-enhancing glioma (NEG) remains challenging. Herein, we analyzed clinical and magnetic resonance imaging (MRI) features to predict malignancy in NEG according to the 2021 WHO classification and developed a clinical score, facilitating risk estimation. A discovery cohort (2012–2017, n = 72) was analyzed for MRI and clinical features (T2/FLAIR mismatch sign, subventricular zone (SVZ) involvement, tumor volume, growth rate, age, Pignatti score, and symptoms). Despite a “low-grade” appearance on MRI, 81% of patients were classified as WHO grade 3 or 4. Malignancy was then stratified by: (1) WHO grade (WHO grade 2 vs. WHO grade 3 + 4) and (2) molecular criteria (IDH^mut^ WHO grade 2 + 3 vs. IDH^wt^ glioblastoma + IDH^mut^ astrocytoma WHO grade 4). Age, Pignatti score, SVZ involvement, and T2/FLAIR mismatch sign predicted malignancy only when considering molecular criteria, including IDH mutation and CDKN2A/B deletion status. A multivariate regression confirmed age and T2/FLAIR mismatch sign as independent predictors (*p* = 0.0009; *p* = 0.011). A “risk estimation in non-enhancing glioma” (RENEG) score was derived and tested in a validation cohort (2018–2019, n = 40), yielding a higher predictive value than the Pignatti score or the T2/FLAIR mismatch sign (AUC of receiver operating characteristics = 0.89). The prevalence of malignant glioma was high in this series of NEGs, supporting an upfront diagnosis and treatment approach. A clinical score with robust test performance was developed that identifies patients at risk for malignancy.

## 1. Introduction

**Figure 1:**
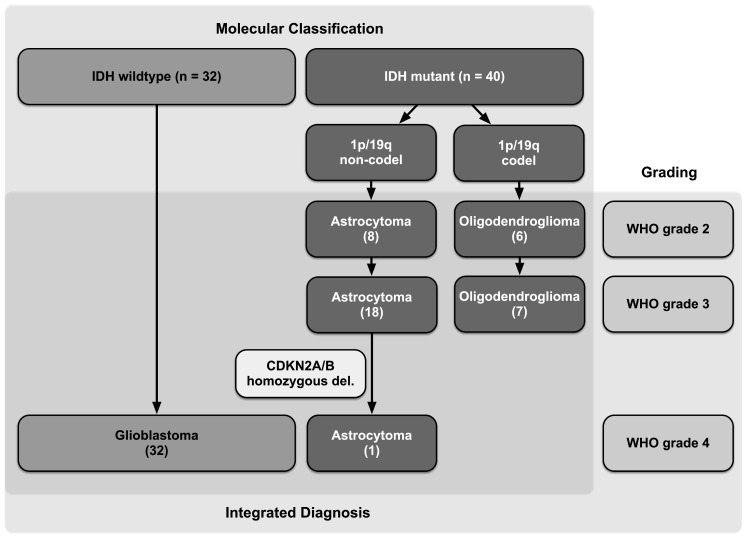
Integrated grading system for glial tumors of the discovery cohort (n = 72), according to the WHO 2021 classification. The combination of histological and molecular grading comprises the integrated diagnosis. Number of patients of the discovery cohort in brackets.

In 2016, the World Health Organization (WHO) introduced a refined classification system of astrocytic and oligodendroglial tumors, combining traditional morphological characteristics with molecular markers such as isocitrate dehydrogenase (IDH) mutation, 1p19q codeletion, and alpha thalassemia/mental retardation syndrome X-linked (ATRX) transcriptional regulator loss to create an “integrated diagnosis” that more accurately predicts prognosis [1,2]. Since IDH mutant (IDH^mut^) astrocytomas have a significantly better prognosis compared to their IDH wildtype (IDH^wt^) counterparts, the term “lower-grade gliomas” has been introduced in clinical practice. Compared to the past, when the term “low-grade glioma” (LGG) was defined by WHO grading alone (WHO grade 2), this term includes IDH^mut^ WHO grade 2 and 3 gliomas (Figure 1) [1,3]. In 2021, the homozygous deletion of cyclin-dependent kinase inhibitor 2A/B (CDKN2A/B) was introduced as another diagnostic hallmark to identify IDH^mut^ astrocytomas with a worse clinical outcome and hence WHO grade 4 classification [4,5]. These changes have somehow facilitated nomenclature, as IDH^mut^ astrocytic gliomas are uniformly classified as astrocytomas and IDH^wt^ are classified as glioblastomas, even when they were formerly graded WHO grades 2 and 3. However, the shift from a pure histological diagnosis towards an integrated molecular diagnosis causes further uncertainty about the optimal timing of diagnosis and treatment in MRI-suspected LGG [1,6,7,8,9]. On one hand, there is growing evidence that upfront, maximized safe resections positively impact the progression-free survival (PFS) and overall survival (OS) of patients diagnosed with LGG [8,10,11,12,13]. On the other hand, a more conservative approach proposes a “watch and wait” strategy, especially in asymptomatic patients or patients with a low tumor burden, delaying histological confirmation of diagnosis until there is MRI or clinical progression [14,15]. While the term “low-grade” suggests a benign pathology, the natural history ultimately terminates in tumor progression, often accompanied by malignant transformation. Therefore, delaying (malignant) progression while minimizing treatment-related complications are the paramount goals. In contrast to LGG, there is scientific consensus that a suspected malignant glioma should be diagnosed and treated upfront. However, the preoperative prediction of malignancy remains challenging, particularly when based on routine MRI. Thus far, imaging parameters, particularly contrast enhancement (CE), have been considered hallmarks for malignancy. A higher grade of uncertainty exists, however, if the tumor does not enhance. Previous studies have pointed out that CE alone cannot be used to assign the tumor a grade in the traditional WHO grading system since up to 40% of WHO grade 3 and 4 gliomas do not enhance [16,17,18]. It can be assumed that the incorporation of molecular markers into the integrated diagnosis has abolished the preoperative assessment of malignancy by CE on MRI. Different approaches attempting to preoperatively assess the IDH mutation status to better delineate IDH^mut^ lower-grade glioma from malignant glioma are underway to make up for this shortcoming. These include advanced MR techniques, such as 2-hydroxyglutarate (2HG) MR spectroscopy [19]. Other groups have analyzed different conventional MR imaging characteristics (tumor size, T2/FLAIR mismatch sign, and growth rate) alone or in combination with classic clinical factors (age) in order to preoperatively predict IDH mutation status [20,21,22,23,24]. Since these data were derived from knowledge generated in the pre-molecular era, we sought to re-evaluate non-enhancing glioma (NEG) in light of the recent update to the WHO classification in 2021 to identify preoperative factors that predict malignancy. In a consecutive series of 72 NEG patients, we first analyzed clinical and MRI factors predicting the WHO grade and IDH mutation status and incorporated the results into a “risk estimation for non-enhancing glioma” (RENEG) score that predicts the presence of a malignant glioma (IDH^wt^ glioblastoma (GBM) and IDH^mut^ astrocytoma WHO grade 4). In a prospective validation cohort of 40 patients, the RENEG scores’ test performance proved to be more robust than the Pignatti score or the T2/FLAIR mismatch sign in detecting a malignant glioma. Therefore, the RENEG score might be helpful in preoperative decision making.

## 2. Materials and Methods

### 2.1. Patient Selection

**Figure 2:**
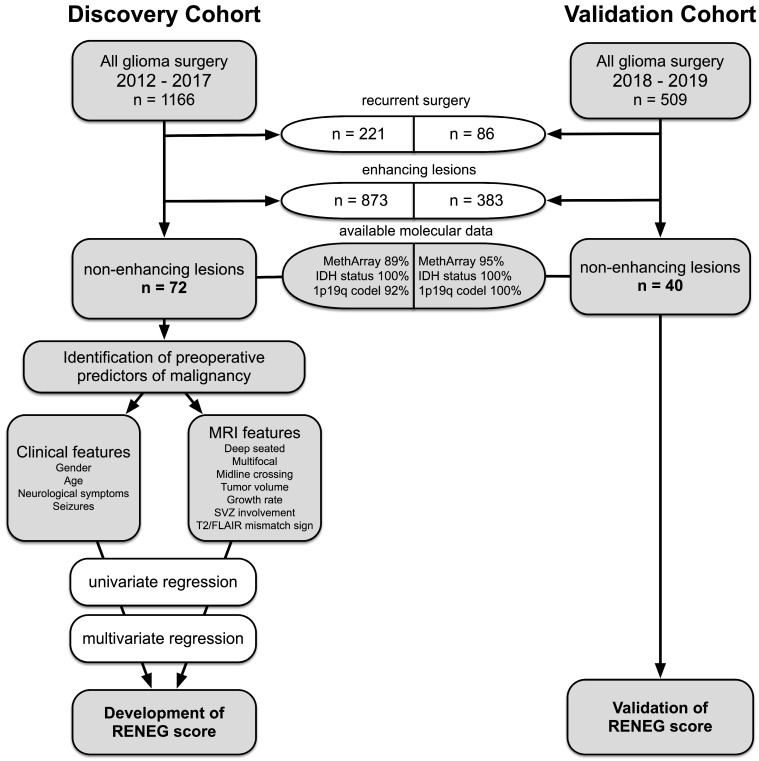
Flowchart of the composition of the discovery and validation sets and the development of the “risk estimation for non-enhancing glioma” (RENEG) score.

We conducted a database search to identify all patients (age ≥ 18 years) who underwent a first supratentorial glioma surgery performed in our department from 2012 to 2017 and for whom preoperative MRI and molecular data were available as part of routine diagnostics (Figure 2). Thus, 1166 consecutive glioma patients were identified. Of these patients, 72 harbored a tumor which was deemed “non-enhancing” on preoperative T1-weighted MR sequences by two independent senior investigators (discovery cohort). For the validation cohort, 509 patients were prospectively screened as they received glioma surgery from 2018 to 2019; of these, 40 patients with non-enhancing lesions were identified. The surgeries included stereotactic or open biopsy and partial, subtotal, and gross total resection. For all cases, medical records were reviewed for clinical information. The Pignatti risk score was assessed as previously described and included the following factors: age, tumor size, tumor crossing the midline, presenting symptoms, and histology [25]. Approval from the ethics committee of the Medical Faculty of the University of Heidelberg was obtained before the initiation of this retrospective study, and patient consent was waived (reference S-005/2003, as of 31 January 2003).

### 2.2. Histopathologic and Molecular Diagnosis

Routine neuropathologic diagnostics were performed in concordance with the 5th version of the WHO classification from 2021 [4]. The following molecular data were obtained as part of daily routine diagnostics: the IDH mutation status was available for all patients of the discovery and validation cohort and was obtained via immunohistochemistry or direct sequencing of the mutation hotspot region [26,27]; 1p/19q codeletion status was available for 66 patients (92%) of the discovery cohort and for all patients of the validation cohort; genome-wide methylation analyses with copy number analyses were available for 89% of the discovery cohort (n = 64) and 95% of the validation cohort (n = 38) and were generated using the Illumina HumanMethylation450 (450k) or Methylation EPIC (850k) array platforms as previously described [28]. For all IDH^mut^ glioma, the CDKN2A/B homozygous deletion status was derived from methylation profiling to identify malignant IDH^mut^ WHO grade 4 astrocytomas that would have otherwise been undergraded.

### 2.3. MRI Evaluation

Sequential MRI was available at initial diagnosis until a few days prior to surgery (“preoperative”). Imaging sequences included standard T1-, T2-, and fluid-attenuated inversion recovery (FLAIR)-weighted sequences. Manual segmentation was performed using the Brainlab™ software SmartBrush version 4.5 (Brainlab, Germany) to quantify tumor volumes on T2- and FLAIR- weighted images in mL at initial and preoperative scans. Tumor growth dynamics (mL/month) were calculated as the difference in tumor volume from the time of initial diagnosis until surgery and were considered a continuous variable in the analysis. The “T2/FLAIR mismatch sign” was defined as previously described by a complete or near complete hyperintense T2 signal and a relatively hypointense signal on FLAIR sequences [23,24]. Further MRI features such as midline crossing (derived from the Pignatti risk score) as well as the involvement of the subventricular zone (SVZ) and multifocality were assessed by a neuroradiologist. The latter two factors were shown to be predictive of poor OS, derived from studies in IDH^wt^ GBM patients [29,30]. We calculated a tumor volume of 27 mL using the formula (a × b × c)/2 for dimensions of a = 6 cm, b = 3 cm, and c = 3 cm to approximate the “largest diameter” of 6 cm used in the Pignatti score. Multifocality was defined when multiple lesions were present without a communicating FLAIR signal. The lesion was considered deep-seated when the insular region or basal ganglia were involved.

### 2.4. Statistical Analysis

The following MRI, clinical, and molecular variables were tested in a univariate logistic regression analysis to identify preoperative predictors of malignancy: T2/FLAIR mismatch sign (yes/no), SVZ involvement (yes/no), preoperative tumor volume (mL; continuous), preoperative growth rate (mL/month; continuous), deep-seated lesion (yes/no), multifocal lesion (yes/no), midline crossing (yes/no), IDH mutation (yes/no), 1p19q codeletion (yes/no), age at histological diagnosis (years; continuous), presenting symptoms (yes/no), and Pignatti risk score (high/low). The univariate analyses were applied for two different classification systems: (1) the traditional WHO grading, distinguishing low-grade (WHO grade 2) from high-grade (WHO grade 3–4) glioma vs. (2) a classification system according to molecular features (IDH^mut^ WHO grade 2 + 3 = “lower-grade” vs. IDH^wt^ GBM + IDH^mut^ astrocytoma WHO grade 4 = “malignant”). The dependent variable was coded with 1 = high grade/malignant and 0 = low/er grade. Thus, odds ratios greater than 1 indicate a positive association of the predictor with a higher probability of malignancy. Variables with regression coefficients most likely deviating from zero (*p* < 0.05) were considered predictors and were included in a multivariate logistic regression model after variable selection. Stepwise regression was performed via the *p*-value. The regression analyses were performed with “R” Software, Version 4.01. 

### 2.5. Development and Validation of a Risk Estimation Score

After variable selection, the estimated coefficients of the regression model were used to derive a risk score approximating the probability of malignancy (the “risk estimation in non-enhancing glioma” (RENEG) score). This score was applied to the validation cohort, and a receiver operating characteristic curve analysis (ROC) was carried out using GraphPad Prism Software (San Diego, CA, USA). The test performance parameters (sensitivity, specificity, positive predictive value (PPV), and negative predictive value (NPV)) were calculated in a four-field table.

## 3. Results

### 3.1. Patient Characteristics of the Discovery Cohort

A total of 72 out of 1166 consecutive glioma patients treated in our department from 2012–2017 underwent surgery for a supratentorial non-enhancing lesion (6.2%). At 46 years, the median age was slightly above the reported age of WHO grade 2 patients, with more males affected than females (m:f = 46:26) [8,11,31]. Most tumors were in the frontal (43%) and temporal (23%) lobes (Table 1). Forty-six patients (64%) were diagnosed because of seizures. Forty-one patients (57%) had preoperative serial MR imaging from which a mean tumor growth rate of 1.22 ± 0.15 mL/month (range 0.11–15.88) was calculated. The mean tumor volume at the time of surgery was 50.8 ± 41.4 mL (range 3–174 mL). A total of 38 tumors (53%) involved the SVZ, and 23 (32%) had a positive T2/FLAIR mismatch sign. Most of the patients received a gross total or subtotal resection, according to an intraoperative or early postoperative MRI (n = 59; 82%). Since all tumors were non-enhancing, resection was guided by FLAIR signal alterations. Adjuvant therapy was administered depending on the integrated diagnosis and initiated in 58 patients (81%). Fourteen patients (19%) did not receive adjuvant treatment. For nine of these patients, a “watch and wait” strategy was pursued, all of them were classified as WHO grade 2. Patients were sub-grouped according to their WHO grade and IDH mutation status, and Kaplan–Meier survival curves were created (Supplementary Figure S1). Since only 10 patients had died by the time of data acquisition, the median OS was not reached and was therefore not considered for outcome analysis. As expected, the median PFS was the shortest for patients harboring IDH^wt^ GBM WHO grade 4 (9 months), whereas WHO grade 2 IDH^mut^ patients had the longest PFS (median PFS not reached). 

### 3.2. High Prevalence of Malignant Gliomas among Non-Enhancing Tumors

IDH mutations were identified in 57% of patients (n = 40; Figure 1). Among these, only 14 patients (35%) were classified as grade 2, and the remaining 26 (65%) were classified as grades 3 and 4. Additionally, 32 patients were diagnosed with an IDH^wt^ GBM. Thus, according to the traditional WHO grading, 81% of all patients (n = 58) with MRI-suspected LGG had tumors classified as malignant (WHO grade 3 or 4), and only 19% (n = 14) had tumors classified as WHO grade 2. Based on the presence of molecular markers, 39 patients (54%) were diagnosed with “lower-grade” gliomas (i.e., WHO grades 2 and 3, IDH^mut^), and 33 patients (46%) were diagnosed with malignant gliomas (i.e., IDH^wt^ GBM WHO grade 4; IDH^mut^ astrocytoma WHO grade 4) (Figure 1, Table 1). Since it has been shown that up to one third of IDH^wt^ patients with tumors formerly classified as WHO grades 2 and 3 did not meet molecular criteria of a GBM according to the new cIMPACT-NOW criteria, these tumors were re-investigated by DNA methylation analysis [32]. The majority of these patients (9/14; 64%) were classified as GBM according to their methylation profile. In three patients, additional methylation analyses could not be performed due to insufficient tumor material, and in two patients, molecular and methylation features were inconclusive and could not be classified into a distinct glioma subgroup. Nevertheless, these two patients presented with a clinical course and treatment regimen comparable to GBM patients and were therefore considered “malignant” for further analyses. Furthermore, to avoid undergrading, O-(2-[^18^F]fluoroethyl)-l-tyrosine positron emission tomography/computed tomography (^18^F-FET PET/CT) scans were performed preoperatively in 17 patients to identify potential “hotspots” for tissue sampling. It is of note that only four ^18^F-FET PET/CT scans (23%) correlated with the final diagnosis, and all of them predicted the presence of a malignant glioma. 

### 3.3. Identification of Preoperative Predictors of Malignancy and Risk Estimation 

**Figure 3:**
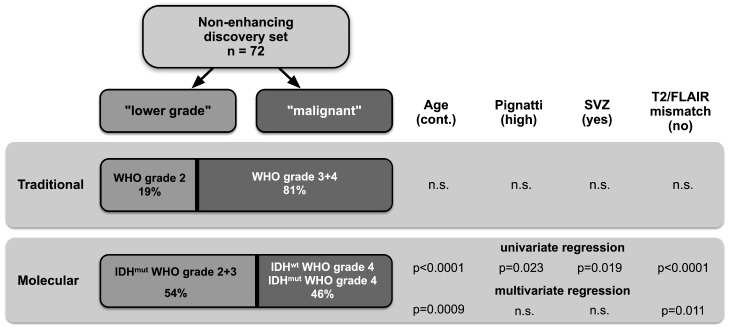
Malignancy was stratified by two different grading systems: (1) traditional grading (WHO grade 2 vs. WHO grade 3 + 4) and (2) molecular grading (IDH^mut^ WHO grade 2–3 vs. IDH^wt^ GBM WHO grade 4 + IDH^mut^ astrocytoma WHO grade 4). Molecular diagnostics allowed clinical and radiographic features to predict malignancy in a more robust manner than traditional grading.

Due to the strikingly high prevalence of malignant gliomas in the discovery cohort, we went on to identify factors predicting malignancy that were derived from routine preoperative MRI and clinical data based on both grading systems: the traditional (with 81% of gliomas classified as malignant) and the molecularly stratified grading system (in which 46% of tumors were still classified as malignant). The following clinical and MRI features were considered for the univariate regression model: gender, age, presence of neurological symptoms or seizures, presence of deep-seated or multifocal lesions, midline crossing, tumor volume, tumor growth rate, SVZ involvement, and T2/FLAIR mismatch sign. We also included the Pignatti risk score (high vs. low), although it incorporates histology [25]. When grouped according to the traditional WHO grading system (WHO grade 2 vs. WHO grade 3 + 4), neither clinical nor MRI features were able to predict malignancy (Table 2, Figure 3). In contrast, when applying the molecularly stratified classification, multiple factors could be identified that distinguished “lower-grade” tumors (IDH^mut^ astrocytoma and oligodendroglioma, WHO grades 2 + 3) from malignant tumors(IDH^wt^ GBM WHO grade 4 + IDH^mut^ astrocytoma WHO grade 4): the Pignatti risk score, SVZ involvement, age, and the T2/FLAIR mismatch sign (*p* = 0.023, *p* = 0.019, *p* < 0.0001, and *p* < 0.0001 respectively). A subsequent multivariate logistic regression after variable selection confirmed age (*p* < 0.001; OR = 1.10 per anno) and the T2/FLAIR mismatch sign (*p* = 0.010; OR = 0.11) to be independent predictive markers of malignancy.

### 3.4. Development of a “Risk Estimation in Non-Enhancing Glioma” Score (RENEG Score)

To facilitate interpretation for clinical decision making, we incorporated the two independent predictive markers “age” and “T2/FLAIR mismatch sign” into a clinical risk score. Since SVZ involvement showed an influence in the univariate analysis and was correlated with PFS (Table 2) we decided to include it in the score as well. The risk function can then be computed from the estimates of the regression model as follows:

βx = −3.727 + 0.165 × SVZ (yes = 1, no = 0) − 2.247 × T2/FLAIR mismatch (yes = 1, no = 0) + 0.089 × age

Then the risk is given by

$$p=\frac{\exp\left(\beta x\right)}{1+\exp\left(\beta x\right)}$$

to harbor a clinically aggressive, “malignant” glioma, i.e., IDH^wt^ GBM WHO grade 4 or IDH^mut^ astrocytoma WHO grade 4 (see www.RENEG.online (accessed on 24 April 2023) for online calculator). According to this calculated probability, the strongest factors for predicting the presence of a malignant glioma represent the absence of the T2/FLAIR mismatch sign (OR = 9.45), followed by age (OR = 2.44/decade) and SVZ involvement (OR = 1.18). 

### 3.5. Validation of the RENEG Score in an Independent Validation Cohort

**Figure 4:**
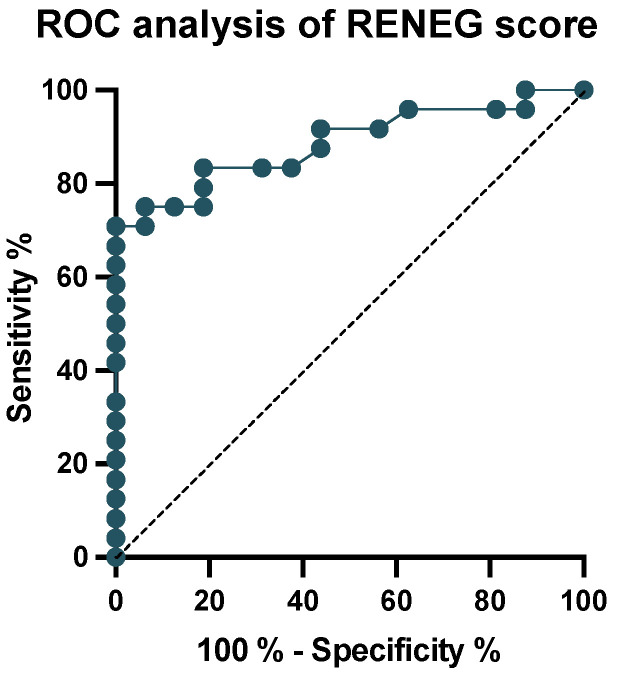
ROC analysis of the RENEG score. The ROC analysis of the RENEG score showed a robust test performance at a cut-off value of 0.59 with an AUC of 0.89 (*p* < 0.0001).

To validate our results, we prospectively analyzed 40 consecutive patients with NEG who received surgery in our department from 2018 to 2019 (Figure 2). The patient characteristics of this validation cohort are shown in Table 1 and are comparable to those of the discovery cohort. The RENEG score was then applied to calculate the likelihood of an individual patient suffering from a malignant (IDH^wt^ GBM WHO grade 4 + IDH^mut^ astrocytoma WHO grade 4) glioma, and the results were used for a ROC analysis (Figure 4). The area under the ROC curve was calculated to be 0.89 (confidence interval = 0.78–0.99; *p* < 0.0001), which is indicative of a good diagnostic performance. Since it is crucial for clinical purposes to identify patients with a malignant glioma and to avoid accidental downgrading, the cut-off value was set to 0.59 to reach a sensitivity of 94% and a specificity of 75% for detecting a malignant glioma. To evaluate if the RENEG score can add value to the widely used Pignatti score or the T2/FLAIR mismatch sign, we calculated sensitivity, specificity, positive and negative predictive values (PPV; NPV), and positive and negative likelihood ratios (LRs) for these two scores in the validation cohort (Table 3). In addition, when comparing the positive LRs to rule in a malignant glioma, our score marked better (3.8) than the Pignatti Score (3.2) and the T2/FLAIR mismatch sign (1.5). This means that a positive test result is obtained 3.8 times more often in patients with a malignant glioma than in patients with a lower-grade glioma (i.e., IDH^mut^ WHO grade 2 or 3). With respect to ruling out a malignant tumor, the negative LR shows an even better value of 0.08, indicatingthat a negative test result is 12.5 times more likely in patients with a lower-grade glioma than in patients with a malignant glioma. The comparative results suggest that the RENEG score may be more reliable than the Pignatti score and the T2/FLAIR mismatch sign in the preoperative detection of high-risk patients with a non-enhancing glioma.

## 4. Discussion

The aim of this study was to identify preoperative clinical and MRI predictors of malignancy according to a molecularly stratified classification system in patients with MRI-suspected “low-grade” non-enhancing gliomas. A higher age as well as the presence of the T2/FLAIR mismatch sign and SVZ involvement were associated with malignancy (i.e., IDH^wt^ GBM WHO grade 4 and IDH^mut^ astrocytoma WHO grade 4) and were thus incorporated into a clinical score for risk estimation.

In our discovery cohort of 72 NEG patients, a surprisingly high proportion was classified as “malignant” in both the traditional (WHO grade 3 and 4; 81%) and molecularly stratified (IDH^wt^ GBM WHO grade 4 and IDH^mut^ astrocytoma WHO grade 4; 46%) classification systems. The strikingly high proportion of malignant tumors in MRI-suspected low-grade glioma patients prompted us to search for clinical and MRI features which could predict malignancy in a preoperative situation. Interestingly, when grouping patients according to WHO grade alone, univariate and multivariate regression models did not reveal a significant association of these features with malignancy, including the factors used for risk estimation by means of the widely used Pignatti score (Figure 3). These results are consistent with a previous study, which suggested that the IDH mutation status may be superior to the Pignatti score in discriminating between low- and high-risk LGG patients [33]. Of note, the Pignatti risk estimation score includes histology and is therefore designed to evaluate the need for adjuvant treatment in a postoperative setting rather than to predict a malignant pathology in the preoperative situation [34]. 

Our study identified age, the T2/FLAIR mismatch sign, and the involvement of the SVZ as preoperative predictors of malignancy. A patient’s age > 40 years has long been considered a risk factor for a poor clinical outcome [25,35]. This strict dichotomic separation has become problematic in light of the new molecular classification, in which a clear cut-off in age was not able to discriminate between molecular entities [31]. To overcome this problem, we included age as a continuous variable in our analysis. 

The T2/FLAIR mismatch sign was first described by Patel et al.; since then, it has been validated by several other groups [22,23,24,36,37]. Its histological correlate is hypothesized to be the formation of microcysts [38]. It is highly specific for IDH^mut^ astrocytoma, but its sensitivity is low, and it is therefore not useful in the clinical routine. In combination with other imaging features, such as calcifications on computer tomography or apparent diffusion coefficient (ADC) or cerebral blood volume (CBV) on MRI, efforts have been made to increase the sensitivity and specificity of non-invasive tests to predict the glial subtype or IDH mutation status but have yielded varying results [39,40]. Additionally, efforts measuring metabolic changes using FET/PET imaging attempt to differentiate between mutational status or malignancy [41,42,43]. In our series, the correlation between FET/PET imaging and integrated diagnosis was low, with an accuracy of 23%. Combining it with further diagnostic modalities such as ADC, which is derived from diffusion imaging and is indicative of tumor areas high in cellularity when reduced, may enhance diagnostic precision [44]. Although ongoing research seems promising, its high operating expense and sparse availability limits its use to centers with high expertise. 

The SVZ represents a distinct area of the adult neurogenic niche and has been repeatedly shown to confer an inferior OS in patients with IDH^wt^ GBM [29,30,45,46,47,48,49]. As SVZ involvement is less frequently found in IDH^mut^ than in IDH^wt^ GBM, we included this feature in our initial prediction marker screen [29,40]. Indeed, it turned out to be a significant factor in the univariate regression model. 

To incorporate all these factors into a useful tool for clinical purposes, we developed a score estimating the risk of a patient with an NEG to harbor a malignant tumor (www.RENEG.online, accessed on 24 April 2023) and validated it in a prospective cohort of 40 patients with NEG who were operated on in our department from 2018 to 2019. The subsequent ROC analysis showed an excellent test performance at a cut-off value of 0.59 and with an AUC of 0.89 (*p* < 0.0001). Two exemplary patients are depicted in Supplementary Figure S2. Finally, to provide perspective on the RENEG score, we compared its results with the Pignatti score and the T2/FLAIR mismatch sign as a standalone marker and calculated the test performance parameters on the validation cohort. It should be noted, however, that the outcome measures of these three tests differ. The Pignatti score incorporates histology as an “invasive” factor accessible only by means of tissue sampling, and tries to identify high-risk patients in need of adjuvant treatment after a diagnosis has been made [25].

The T2/FLAIR mismatch sign has been identified as a radiographic characteristic of IDH^mut^ astrocytoma regardless of grading. Nevertheless, while differing in outcome measures, all scores are set out to identify patients at risk of a poor outcome. In this regard, the RENEG score performed best in “ruling in” a malignant glioma (>0.59: LR+ 3.75) and even better in “ruling out” a malignant glioma (>0.59: LR− 0.08). The ultimate goal of this score is to preoperatively identify patients with newly diagnosed NEG for whom upfront tissue sampling and/or resection should be advocated without further watchful waiting.

We do acknowledge the limitations of this study. Most importantly, the results of this study will have to be validated in an external cohort, preferably in a multi-institutional study, to account for variations in the study parameters such as MRI scanning parameters or interobserver variations. Furthermore, in both the discovery and validation cohorts, we included resected and biopsied patients. Even with additional diagnostic tools such as FET-PET, we cannot exclude that patients undergoing open or stereotactic biopsies are subject to sampling bias. However, most diagnoses of our cohort were based on a methylation array analysis. Wenger et al. reported that in a series of 12 GBM patients, each with spatially different tumor biopsies, although intratumoral DNA methylation was heterogeneous, all tissue samples were uniformly classified as GBM IDH wildtype or mutant [50]. Therefore, regarding the development of our score, the sampling bias should be low. 

## 5. Conclusions

The prevalence of malignant glioma was unexpectedly high in this series of molecularly characterized non-enhancing gliomas despite their “low-grade” MRI appearance, questioning the widely used concept of watchful waiting in these patients and advocating for early surgical intervention whenever feasible. To optimize preoperative risk estimation, we developed the RENEG score, which incorporates basic clinical and MRI factors to predict malignancy with a high diagnostic accuracy and easy clinical use. 

## Figures and Tables

**Table 1:** Patient characteristics of the discovery and validation cohorts.

	Discovery Cohort n = 72	Validation Cohort n = 40
**Patient characteristics**		
Age at first diagnosis (mean ± SD; range)	46 ± 16 (20–77)	49 ± 16.6 (14–78)
Gender (female: male)	26: 46	22: 18
Mean follow-up (months ± SD; range)	25 ± 19 (1–127)	26 ± 35 (0–137)
Mean OS (months ± SD; range)	20 ± 13 (2–45)	not reached
Mean PFS (months ± SD; range)	14 ± 17 (0–67)	not reached
Progression (% of total)	30 (43%)	16 (40%)
Deaths (% of total)	9 (13%)	0 (0%)
**Symptoms**	(% of total)	
Seizures	46 (64%)	18 (45%)
Headache	10 (14%)	3 (8%)
Incidental finding	7 (10%)	9 (23%)
Vertigo	5 (7%)	0 (0%)
Motor deficits	4 (6%)	5 (13%)
Psychological disorder	4 (6%)	2 (5%)
Unspecific symptoms	2 (3%)	3 (8%)
**Radiographic characteristics**		
Patients with preop follow-up MRI (% of total)	40 (57%)	
Tumor volume (mL) at first diagnosis (mean ± SD; range)	47.1 ± 41.0 (3–174)	
Tumor volume (mL) preop (mean ± SD; range)	50.8 ± 41.4 (3–174)	
Mean growth rate (ml/month ± SD; range)	1.22 ± 0.15 (0.1–15.9)	
SVZ involvement (% of total)	38 (53%)	25 (63%)
T2/FLAIR mismatch sign positive (% of total)	23 (32%)	8 (20%)
**Localization**		
frontal lobe	32%	
temporal lobe	31%	
parietal lobe	3%	
other	6%	
**Molecular diagnostics**	(% of total)	
IDH mutation	41 (57%)	24 (60%)
1p19q codeletion	13 (18%)	12 (30%)
CDKN2A/B homozygous deletion	9 (12.5%)	6 (15%)
**Surgical procedure**	(% of total)	
Gross/subtotal resection	53 (73%)	22 (55%)
Biopsy	19 (26%)	18 (45%)
**Adjuvant treatment according to integrated diagnosis**	58 of 72 (81% of total)	28 of 40 (70% of total)
Astro IDH^mut^ WHO grade 2	6 of 14 (42%)	5 of 11 (45%)
Astro IDH^mut^ WHO grade 3	24 of 25 (96%)	1 of 1 (100%)
Astro IDH^mut^ WHO grade 4	1 of 1 (100%)	-
Glioblastoma IDH^wt^ WHO grade 4	27 of 32 (84%)	15 of 16 (94%)
Oligo IDH^mut^, 1p19q codel WHO grade 2	4 of 6 (67%)	6 of 11 (55%)
Oligo IDH^mut^, 1p19q codel WHO grade 3	6 of 7 (86%)	1 of 1 (100%)
no adjuvant treatment	14 (19% of total)	12 (30% of total)

**Table 2:** Identification of clinical and MRI factors discriminating low(er)-grade from malignant glioma depending on the classification system in univariate and multivariate regression analyses (OR > 1 likelihood of malignant).

	WHO Grading			Molecular Classification				
	WHO Grade 2 and 3 vs. WHO Grade 4			IDH^mut^ WHO Grade 2 and 3 vs. IDH^wt^ and IDH^mut^ WHO Grade 4				
	Univariate			Univariate			Multivariate	
	OR	95% CI	*p*-Value	OR	95% CI	*p*-Value	OR	*p*-Value
**Clinical features**								
Gender (m)	0.85	0.26–2.56	0.777	0.84	0.32–2.21	0.723		
Age (cont.)	1.02	0.99–1.06	0.240	1.12	1.07–1.18	**<0.0001**	1.10	**0.0009**
Specific symptoms ^a^ (yes)	1.00	0.28–3.18	1.0	1.13	0.4–3.25	0.539		
Seizure (yes)	1.27	0.41–3.81	0.669	0.95	0.36–2.54	0.815		
Pignatti risk (high)	0.61	0.14–2.22	0.468	6.5	1.51–44.95	**0.023**		
Age (>40)	2.33	0.78–7	0.132	10	3.34–34.76	**<0.0001**		
Volume > 28 mL (yes)	0.92	0.3–2.73	0.890	0.46	0.17–1.19	0.114		
Neurological deficit ^b^ (yes)	0.64	0.11–4.9	0.625	2.4	0.44–18.18	0.331		
Midline crossing (yes)	0.39	0.11–1.49	0.153	2.62	0.74–10.69	0.149		
Astrocytoma (yes)	3.33	0.93–12.05	0.060	- ^c^		- ^c^		
**Radiographic features**								
Deep seated (yes)	1.39	0.31–9.86	0.695	3.03	0.76–15.06	0.133		
Multifocal (yes)	0.64	0.11–4.92	0.625	1.19	0.2–6.49	0.887		
Volume at first diagnosis (cont.)	1.00	0.99–1.01	0.762	1.00	0.99–1.01	0.669		
Volume at surgery	1.00	0.99–1.01	0.894	1.00	0.99–1.01	0.685		
Growth rate (mL/d)	6.94	0.05–2016.8	0.454	14.3	0.2–4206.21	0.258		
SVZ involvement (yes)	2.12	0.72–6.56	0.177	3.23	1.24–8.7	**0.019**		
T2/FLAIR mismatch sign (yes)	1.30	0.42–4.56	0.662	0.05	0.01–0.2	**<0.0001**	0.11	**0.011**

^a^ symptoms considered unspecific: headache, vertigo; ^b^ according to Medical Research Council neurologic scale; ^c^ no reasonable estimate possible because all patients in highly graded group had astrocytoma; m = male; OR = odds ratio; CI = confidence interval; cont. = continuous; SVZ = subventricular zone.

**Table 3:** Test performance parameters in detecting “high risk“ patients (RENEG score—malignancy as defined by IDH^wt^ GBM WHO grade 4 + IDH^mut^ astrocytoma WHO grade 4; Pignatti score—“high risk“ patients; T2/FLAIR mismatch—IDH^wt^ glioma).

	RENEG Score (Cutoff Value: >0.59)	Pignatti Score (High Risk)	T2/FLAIR Mismatch (Absent)
Sensitivity	0.94	0.57	0.67
Specificity	0.75	0.82	1.00
PPV	0.71	0.81	1.00
NPV	0.95	0.58	0.67
LR+	3.75	3.20	1.5
LR−	0.08	0.53	0

PPV = positive predictive value; NPV = negative predictive value; LR +/− = positive/negative likelihood ratio.

## Data Availability

The data presented in this study are available in this article.

## Supplementary Materials

The following supporting information can be downloaded at: https://www.mdpi.com/article/10.3390/cancers15092503/s1, Supplementary Figure S1: Kaplan–Meier progression-free-survival (PFS) curves of the 72 patients of the discovery cohort. Supplementary Figure S2: Two exemplary patients for whom the RENEG score is applied.
