# Supplementary material for: Risk Estimation in Non-Enhancing Glioma: Introducing a Clinical Score
Source: Cancers (Basel). 2023 Apr 27;15(9):2503. doi: 10.3390/cancers15092503 (PMC10177456; doi:10.3390/cancers15092503)
Supplement: Supplementary file 1 [file cancers-15-02503-s001.zip › cancers-2292226-supplementary.pdf]

**Supplementary Figures**

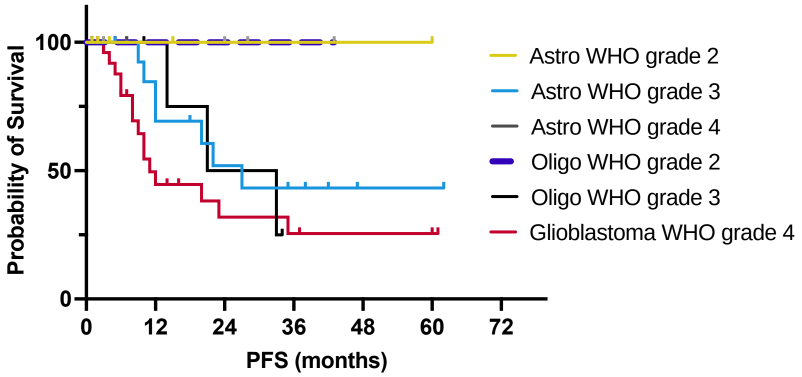


**Figure S1:** Kaplan Meier progression-free-survival (PFS) curves of the 72 patients of the discovery cohort. As expected IDH$^{wt}$ GBM patients WHO grade 4 had the shortest PFS, whereas IDH$^{mut}$ astrocytoma WHO grade 2 patient had the longest PFS.

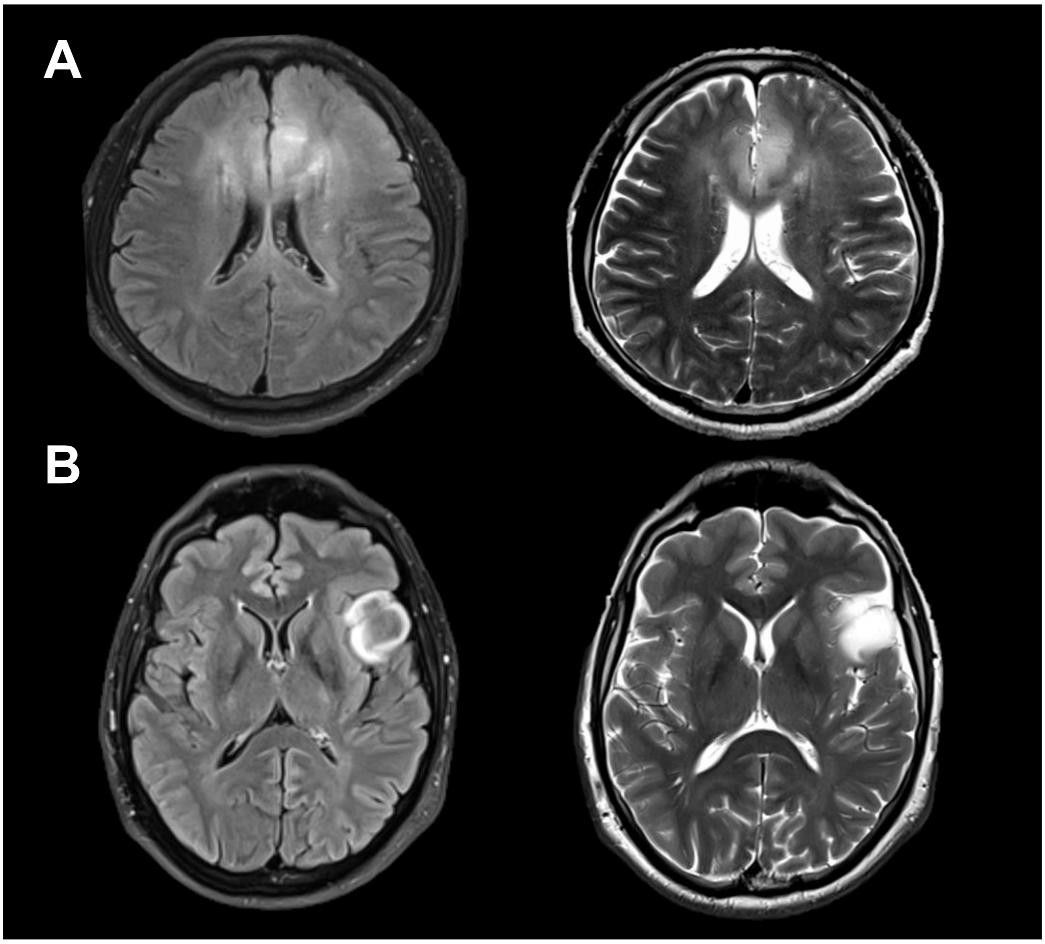


**Figure S2.** Two exemplary patients where the RENEG score is applied. **A.** 46-year-old male patient with an incidental bifrontal non-enhancing FLAIR lesion. A watch and wait strategy was adopted and the lesion was observed for three years until MRI progression. It involved the SVZ and had no T2/FLAIR mismatch sign therefore yielding a RENEG score of 0.61 making the lesion very likely to be classified as malignant. Indeed, postoperative histology revealed an IDHwt GBM WHO grade 4. **B.** 54-year-old male patient presenting with headaches. The frontal lesion was positive for the T2/FLAIR mismatch sign and did not contact the SVZ. Therefore, the calculated RENEG score is 0.19, making it very unlikely of being malignant. This was confirmed by histology (IDHmut astrocytoma WHO grade 2).
